# Adult-Onset Still's Disease: From Pathophysiology to Targeted Therapies

**DOI:** 10.1155/2012/879020

**Published:** 2012-06-26

**Authors:** Clio P. Mavragani, Evangelos G. Spyridakis, Michael Koutsilieris

**Affiliations:** Department of Experimental Physiology, School of Medicine, University of Athens, Athens 11527, Greece

## Abstract

Adult-onset Still's disease (AOSD) is a systemic inflammatory disorder affecting primarily young individuals. The diagnosis is primarily clinical and necessitates the exclusion of a wide range of mimicking disorders. Given the lack of solid data in regard to the underlying pathogenetic mechanisms, treatment of AOSD has been for years largely empirical. Recent advances have revealed a pivotal role of several proinflammatory cytokines such as tumor necrosis factor-**α** (TNF-**α**), interleukin-1 (IL-1), interleukin-6 (IL-6), interleukin-8 (IL-8), and interleukin-18 (IL-18) in disease pathogenesis, giving rise to the development of new targeted therapies aiming at optimal disease control.

## 1. Introduction 

Adult-onset Still's disease (AOSD) is a rare inflammatory disease of unknown etiology, which commonly affects young adults. It is usually characterized by high spiking fevers, arthritis, and an evanescent, nonpruritic, macular and salmon coloured rash, appearing on the trunk and the extremities. Organomegaly, lymphadenopathy, serositis, and aseptic meningitis can also occur. Important laboratory findings include leukocytosis, with predominance of neutrophils, negative testing for rheumatoid factor (RF), and antinuclear antibodies (ANA) as well as high serum ferritin levels and low serum glycosylated ferritin levels [1–3].

Severe disease complications include pericarditis, endocarditis, haemolytic anaemia, and macrophage activation syndrome (MAS). The latter is characterized by thrombocytopenia, markedly elevated ferritin levels, hypofibrinogenemia, and elevated aspartate amino-transferase (AST). AOSD diagnosis can be safely established, after important mimickers including infections, malignancies, and autoimmune diseases are excluded. Treatment of patients with AOSD includes nonsteroidal anti-inflammatory drugs (NSAIDs), corticosteroids, and disease-modifying antirheumatic drugs (DMARDs), while our better understanding of disease pathophysiology allowed the identification of biological agents as important targeted therapies [1, 4]. 

Recent studies have added valuable information in regard to the underlying pathogenetic mechanisms of AOSD. Besides, the exact pathogenesis remains largely elusive, with genetic, environmental, and immunologic contributors being implicated. In the present paper, we aimed to summarize recent advances in pathophysiology and potential therapeutic strategies in the setting of AOSD. 

## 2. Methods

 We conducted a MEDLINE database search using the Pubmed interface. We also used rheumatology textbooks with chapters relevant to AOSD and abstract database from ACR and EULAR meetings at 2010 and 2011. We searched for “Adult onset Still's disease and biological agents,” “AOSD or Still's disease pathophysiology,” “AOSD or Still's disease therapy,” “Still's disease or AOSD treatment,” “Still's disease or AOSD and inflammasome.”

## 3. Etiopathogenesis

### 3.1. Genetics

Several small case studies have previously reported associations with distinct HLA alleles in patients with AOSD, with often conflicting results. In an early small study of 25 AOSD patients, HLA-Bw35 was associated with disease susceptibility conferring a favourable prognosis [5]. Wouters et al. reported an increased frequency of the HLA-DR4 allele in 29 patients with AOSD compared to normal controls, with the presence of HLA-DRw6 being linked to root joint involvement [6]. In a subsequent study, a strong disease association with HLA-B17, B18, B35, and DR2 has been documented [7]. In Japanese populations, an association between a chronic articular form of AOSD and HLA-DRB1*1501 (DR2), DRB1*1201 (DR5), and DQB1*0602 (DQ1) was previously reported, while HLA- and DQB1*0602 (DQ1) have been also associated with the systemic form of the disease [8]. Data from a Korean report, supported an association between HLA-DRB1*12 and DRB1*15 and AOSD, while HLA-DRB1*04 seemed to be protective. On the other hand, HLA-DRB1*14 alleles were more commonly present in patients with the monocyclic systemic type of AOSD [9]. 

### 3.2. Infections

The shared clinical and laboratory findings observed in AOSD and infections are highly suggestive of a putative role of infectious agents in disease pathogenesis. Several anecdotal reports so far indicate a temporal relationship between bacterial and viral triggers prior to disease onset. Several viruses such as rubella, Echovirus 7, mumps, cytomegalovirus (CMV), and others, as well as bacterial pathogens including Yersinia enterocolitica, Chlamydophila pneumoniae, Brucella abortus, and Borrelia burgdorferi, have been so far implicated in disease pathogenesis [10–13]. However, to date definite clue for their precise role is lacking. 

## 4. Pathophysiology

### 4.1. Cellular Populations

#### 4.1.1. Innate Immunity

A hallmark of AOSD is neutrophil and macrophage activation possibly under the effects of the proinflammatory interleukin-18 (IL-18) signalling. Neutrophil (PMN) CD64 a marker of neutrophil activation has been recently found to be upregulated in patients with active AOSD [14]. A calcium-binding protein named calprotectin, secreted by activated neutrophils and macrophages, as well as macrophage migration inhibitory factor (MIF), is useful markers of disease activity and severity [15, 16]. Intercellular adhesion molecule-1 (ICAM-1) upregulated by IL-18- has been also proposed as a potential clinical marker, as its expression typically reflects the level of disease activity [17]. Furthermore, activation and differentiation of macrophages appears to be orchestrated by macrophage-colony stimulating factor (M-CSF), a cytokine which is substantially elevated in acutely ill AOSD patients [18]. 

#### 4.1.2. Adaptive Immunity

The role of CD4^+^ T helper (Th) cells in the pathogenesis of AOSD has been recently appreciated, with Th1 subset predominating over that of Th2 CD4^+^ cells and being associated with disease activity. Accordingly, interferon-gamma (IFN-*γ*) mRNA expression was found to be significantly higher than that of interleukin-4 (IL-4) in skin and synovial tissue biopsies [19].

The role of Th17 lineage in AOSD pathogenesis is also emerging, as evidenced by increased number of peripheral Th17 cells in 24 patients with untreated and active AOSD compared to healthy controls [20]. Th17 cells are a subset of T helper cells, named after their ability to produce interleukin-17 (IL-17). This subset of cells is derived from the differentiation of naive CD4^+^ T cells, under the influence of transforming growth factor *β* (TGF*β*), interleukin-1*β* (IL-1*β*), and interleukin-6 (IL-6) [20, 21]. Interestingly, heightened levels of T-cell receptor *γδ*-positive (TCR*γδ*+) T cells, mostly of the V*γ*9/V*δ*2 subset have been previously associated with active disease and correlated with inflammatory markers [22]. Since, it has been recently appreciated that T*γ*/*δ* cells are also represent an important source of IL-17 production, the role of these cells in the pathogenesis of AOSD requires further attention [23]. 

Interleukin-18 (IL-18), interleukin-23 (IL-23), and inteleukin-21 (IL-21)—found to be elevated in active AOSD patients—seem also to ensure the proliferation/maintenance of Th17 cells. Circulating Th17 cells correlated with disease activity and ferritin as well as IL-1*β*, IL-6, IL-17, IL-18, IL-21, and IL-23 levels. Of interest, Th17 numbers were normalized after successful therapy [20]. 

Additional T cell populations actively involved in AOSD pathogenesis include the CD4^+^ CD25 (high) T regulatory (Treg) cells found to be low in these patients compared to healthy controls and inversely associated with disease activity. Furthermore, higher levels of CD4^+^ CD25 (high) Treg cells have been associated with a more favourable prognosis, as patients with monocyclic disease, a mild form of AOSD, typically have higher concentrations of circulating CD4^+^ CD25 (high) Treg cells than those with polycyclic or chronic articular form [24]. 

### 4.2. Cytokines/Chemokines

Several cytokines and chemokines have been so far implemented in the pathogenesis of AOSD. It should be, however, pointed out that according to recent findings, cytokine profile has not been proven useful in differentiating patients with AOSD from those with sepsis, limiting their potential use in clinical practice [25]. 

### 4.3. Cytokines (Table 1)

#### 4.3.1. Tumor Necrosis Factor-*α* (TNF-*α*)

Increased TNF-*α* levels were detected in sera and tissues from AOSD patients compared to healthy controls independently of disease activity. On the other hand, serum levels of soluble tumor necrosis factor-receptor-2 (sTNF-R2) correlated with serum CRP levels, implying its potential use as a disease activity marker [8, 26, 30]. 

#### 4.3.2. Interleukin 1 (IL-1)

 IL-1 appears to be implicated in AOSD pathogenesis as its serum concentration is elevated in these patients compared to healthy controls. Further evidence for the contribution of IL-1 in AOSD pathophysiology came from the pioneering work by Pascual et al. reporting that incubated peripheral blood mononuclear cells (PBMCs) with serum from patients with systemic form of juvenile idiopathic arthritis (SJIA), led to increased expression of innate immunity genes and release of large amounts of IL-1*β* [27]. However, p*ο*lymorphisms in the IL-1*β* and IL-1 receptor (IL-1R) genes have not been associated with AOSD susceptibility, at least in a Korean population [37]. Recent findings suggest activation of the protein complex nucleotide-binding oligomerization-domain-(NOD-) like receptor family, pyrin domain containing 3 (NLRP3) inflammasome, as an important source of IL-1*β*; this activation can occur by recognition of pathogen-associated molecular patterns (PAMPs) and danger-associated molecular patterns (DAMPs). Although it seems to contribute at least in one subset of SJIA—the pediatric counterpart of AOSD—with a favorable response to IL-1 blockade, further studies are required to fully explore its exact role in the pathogenesis of AOSD [38–40]. Taken together, these observations may suggest that susceptibility to SJIA and AOSD might be conferred by an interplay with exogenous pathogens-triggers of inflammasome with genetically determined inflammasome responsiveness resulting in dysregulation of IL-1*β* production [41]. Further studies are required to delineate these processes.

#### 4.3.3. Soluble Interleukin-2 Receptor (sIL-2R)

Heightened  sIL-2R levels, a marker of T-cell activation, were also reported in two distinct studies of AOSD patients, serving as a potential marker of disease activity [8, 29]. 

#### 4.3.4. Interleukin-6 (IL-6)

IL-6 levels have been found to be elevated in AOSD patients compared to their healthy counterparts in association with disease activity, fever spikes, and CRP levels. Of interest, skin lesional biopsies from individuals presenting with the characteristic salmon coloured rash revealed heightened IL-6 levels [29–31]. In addition, IL-6 may contribute to the increased levels of ferritin as it stimulates its production along with CRP and other acute-phase proteins by the liver [26]. Finally, prolonged exposure to high levels of IL-6 may be associated with severe growth impairment, especially in patients with SJIA [42].

#### 4.3.5. Interleukin-8 (IL-8)

IL-8, a proinflammatory cytokine, which mobilizes, activates, and degranulates neutrophils at the site of inflammation has been also found to be raised in AOSD patients compared to healthy controls, independently of activity status [29]. Given that elevated levels of serum IL-8 typically characterize the chronic articular form of AOSD, they can be used as a marker to predict the persistence of arthritic complaints [30].

#### 4.3.6. Interleukin-17 (IL-17)

As previously mentioned and in line with previous observations in other autoimmune diseases [43], serum IL-17-*α* proinflammatory cytokine derived by Th17 cells was higher in patients with AOSD and correlated with Th17-circulating cells. The fact that Th17 cells and IL-17 levels were both abated upon therapy administration implies a potential therapeutic role of Th17 targeted therapies in the management of those diseases [20].

#### 4.3.7. Interleukin-18 (IL-18)

IL-18-*α* member of the IL-1 family, which induces Th1 cytokine production-[44], has been shown to be higher in the serum synovial tissue and lymph nodes in patients with AOSD than in healthy individuals, serving as a marker of disease severity, possible response to corticosteroids and of AOSD-related hepatitis [29, 32, 33, 45]. The latter is evidenced by the demonstrated association of IL-18 serum levels with active liver disease. Locally rather than systematically produced IL-18 by liver activated macrophages (CD68^+^) seems to contribute to this complication [30, 34]. Associations of IL-18 with serum ferritin, C-reactive protein (CRP), and neutrophil count have been also demonstrated [8, 34, 35]. Several polymorphisms of the IL-18 gene have been associated with AOSD in Japanese and Chinese populations [46–48]. 

Another function attributed to IL-18 is that of lymphocyte apoptosis possibly through induction of Fas Ligand (FasL) and p53 pathways, both implicated in the programmed cell death [49]. This hypothesis is also supported by raised Fas and FasL levels in untreated AOSD patients compared to healthy controls [50]. 

Finally, in a more recent report, IL-18 levels were found to be significantly elevated in patients of AOSD complicated by MAS compared to M-CSF levels; an opposite observation was made in patients with lupus-associated MAS [36]. 

#### 4.3.8. Interferon-Gamma (IFN-*γ*)

Although IFN-*γ* levels were also found to be raised in AOSD patients compared to healthy individuals, no study so far demonstrated association of this cytokine with disease activity [26, 29]. 

### 4.4. Chemokines

The contributory role of chemokines in the pathophysiology of AOSD was supported by a recent study reporting elevated levels of CX3CL1, CXCL8, CXCL10, CCL2, and CCL3 in serum of AOSD patients compared to healthy controls. Of interest, only CX3CL could be used as a marker of disease activity as it was correlated well with serum CRP, ferritin, IL-18, and sIL-2R levels. Furthermore, markedly elevated concentration of CX3CL1 and ferritin was able to predict the onset of MAS, indicating its value in predicting AOSD-related complications [51].

## 5. Cytokines as Therapeutic Targets (Table 2)

Treatment of patients with AOSD has been empirical for a long time, given the lack of solid data from well-designed double-blinded randomized clinical trials with the majority of evidence deriving from small case series and retrospective studies [66]. Recent advances in better understanding of disease pathophysiology allowed the designation of targeted therapies leading to effective disease control [67]. Conventional immunosuppressants and new biologics are the main agents included in our therapeutic armamentarium against AOSD.

While nonsteroidal anti-inflammatory drugs (NSAIDs) have been previously considered as a first-line medication for the treatment of AOSD, they have been replaced by corticosteroids, as they are effective as monotherapy only in 7–15% of patients [66, 68]. The steroid therapy is efficacious in approximately two thirds of patients and more pronounced among those without chronic articular disease [69]. Disease-modifying antirheumatic drugs (DMARDs) such as methotrexate (MTX), cyclosporine, hydroxychloroquine, gold, penicillamine, and azathioprine, [70–72] have been proven efficacious in steroid-resistant or -dependent AOSD cases, with methotrexate being the most commonly used DMARD in clinical practice with response rates up to 60% [69]. In regard to sulfasalazine, reduced efficacy along with some previously raised safety issues given the reported associations with MAS development discourages its use in AOSD cases [73–75]. In patients refractory to treatment with steroids and/or DMARDS, biological agents seem to achieve a better control of disease activity.

Despite the lack of solid evidence of TNF implication in the pathogenesis of AOSD as opposed to rheumatoid arthritis, anti-TNF agents have been used in AOSD refractory cases with modest success, particularly in the chronic articular form of the disease lagging in efficacy behind IL-1 and IL-6 inhibitors. In a small case series of twelve AOSD cases refractory to DMARDs, administration of etanercept,a soluble TNF receptor, led to arthritis improvement in 7 patients with nonsignificant adverse events [53]. Infliximab, a monoclonal antibody against TNF*α*, as a treatment of eight multidrug-resistant AOSD cases led to full response in 87.5% (7/8) of patients. Five of these patients remained in remission even after the discontinuation of infliximab and one of them switched to etanercept due to infusion reactions. Only one of the responders required chronic therapy to control its arthritis and only one patient did not respond to these biological agents [54]. In two additional cases series, infliximab was administrated along with corticosteroids and DMARDs in a small number of patients with remission of systemic features, normalization of inflammatory markers, and without serious adverse reactions [52, 55].

Further information regarding the safety and the efficacy of anti-TNF*α* agents derives from a study published by Fautrel et al. in which infliximab or etanercept was administrated to twenty AOSD patients, five with systemic and fifteen with polyarticular form, whose response to MTX and corticosteroids was considered inadequate. The majority of patients responded partially to therapy (64%, or 16 of 25 patients) and only five in twenty patients achieved complete remission [56]. Anti-TNF-*α*-induced cutaneous adverse effects in the setting of SJIA have been reported including cutaneous vasculitis and lichen planus, as well as psoriatic palmoplantar pustulosis accompanied by plaque-type psoriasis localized to the scalp [76].

In view of the central role of IL-1 in pathogenesis of AOSD as previously reported, administration of interleukin-1 receptor antagonist (anakinra) in these patients seems a logical approach [28]. In a retrospective analysis of 25 AOSD patients, it has been shown that patients (84%) receiving anakinra either as monotherapy or as adjunct therapy responded completely within a few days and only one of them had its disease relapsed during the subsequent followup. The remaining patients experience a partial clinical (12%) and laboratory (16%) response and only three patients discontinued the drug because of adverse effects. In general, the need for corticosteroids during treatment with anakinra greatly diminished in every patient [61]. The corticosteroid-sparing effect of anakinra along with its effectiveness was also noted in a case series reported by Kalliolias et al. in 2007 [59]. Furthermore, Fitzgerald et al. demonstrated that anakinra is an effective agent to treat AOSD patients refractory to corticosteroids, MTX and etanercept, as this drug rapidly resolves the inflammatory response and leads to normalization of laboratory markers [57]. 

Moreover, Lequerré et al., in a study including both AOSD and SJIA patients, suggested anakinra as an effective alternative in the treatment of patients with AOSD, with somewhat limited efficacy in SJIA population [60]. In contrast, in a retrospective chart review of 46 SJIA patients, receiving initially anakinra either as monotherapy or together with additional disease-modifying antirheumatic drugs revealed that in 60% of these patients the clinical activity resolved completely and laboratory markers were normalized. A full response was also achieved in 80% of patients receiving anakinra as monotherapy. The authors concluded that anakinra should be considered a safe and an effective way not only to treat systemic SJIA but also to prevent the emergence of intractable arthritis [62]. In addition, according to a case report published by Raffeiner et al., anakinra could be successfully used in the treatment of a patient with AOSD and myocarditis [77]. On the other hand, Ruiz et al. reported that anakinra could not prevent the progression of AOSD-associated cardiac disease despite the excellent control of noncardiac symptoms of a patient, without excluding the possibility that anakinra may be implicated in cardiac events of this patient [78].

Although anakinra seems to be an effective treatment of AOSD patients regarding the rapid resolution of their clinical and laboratory markers, a recent case report published by Lahiri and Teng has shown that joint damage may progress despite the administration of this drug [79]. A second generation IL-1 inhibitor, the IL-1 trap rilonacept, has been used in 3 patients who had failed treatment with glucocorticoids, immunosuppressors, and biologics, including anakinra with promising results [80]. Furthermore, according to a more recent work reported by Kontzias et al., canakinumab, a fully human monoclonal antibody against IL-1*β* with a long half-life, successfully controlled disease flares in AOSD patients refractory to DMARDs, anakinra (short-acting IL-1 blockade), and rilonacept (moderate-acting IL-1 blockade). The pharmacokinetic properties of anakinra may account for its relative ineffectiveness compared to canakinumab [65]. In addition, the efficacy and safety of canakinumab in the treatment of SJIA, the pediatric counterpart of AOSD, has been demonstrated in a phase II, multicenter, open-label study, with 60% of patients achieving an ACR Pediatric 50 response [64].

Given the emerging role of T-cells in the pathogenesis of AOSD, administration of abatacept, a T-cell costimulation modulator, in these patients seems to be a logical approach. Abatacept (CTLA4IgFc), a fusion protein which consists of the extracellular domain of the cytotoxic T-lymphocyte antigen 4 (CTLA-4) and the Fc portion of immunoglobulin G1 (IgG1), inhibits T-cell activation by binding to CD80 and CD86 receptors on antigen-presenting cells (APCs) and preventing their interaction with CD28 receptor on T cells. Recent findings support a potential role of the latter in AOSD cases refractory to conventional DMARDs, anti-TNF-*α* agents, and even to IL-1 receptor antagonists [81, 82]. 

Given that IL-6 shares an important pathogenetic role in AOSD, as mentioned above, the interleukin-6 (IL-6) antagonist, tocilizumab (TOC), has recently been proposed as a potential treatment for these patients. Indeed, it seems to be an effective drug even against AOSD cases refractory to anakinra and TNF-*α* antagonists in anecdotal cases, even as monotherapy [83–91]. Tocilizumab was also able to control disease activity in a patient with diffuse intravascular coagulation (DIC) and AOSD, refractory to cyclosporine and high-dose glucocorticoids. In addition, the dose of corticosteroids was greatly reduced as TOC was added on maintenance therapy [92]. On the other hand, MAS seemed to follow TOC administration in a patient with intractable AOSD, implying that caution should be taken in very active forms of the disease [93]. In the first case series of tocilizumab in fourteen patients with intractable AOSD at a dose 5–8 mg/kg every two or four weeks, eleven patients completed the 6-month followup and the remaining three discontinued the drug due to adverse effects, including necrotizing angiodermatitis, infusion-related chest pain, and systemic flare. Over the course of 6 months, the clinical activity resolved completely in 57% of patients (8/14) and corticosteroid maintenance dose was dramatically reduced, suggesting that TOC may be an effective alternative treatment, when dealing with multidrug-resistant cases of AOSD [63]. TOC has also been approved for the treatment of SJIA patients, as it is associated with substantial clinical and laboratory responses [58]. Of interest, administration of this drug in SJIA patients led to improvement of reduced serum cartilage oligomeric matrix protein (COMP), further supporting the concept of contribution that high levels of IL-6 in the suppression of growth cartilage turnover [42, 94]. 

## 6. Conclusion

Taken together, these findings support the contributory role of several immune mediators in AOSD pathogenesis allowing the determination of rational treatment approaches. While current evidence identifies IL-1 blockade as a major therapeutic strategy in patients with refractory AOSD, inhibition of IL-6, IL-17, or IL-18 molecules holds significant promises. Given the complex and multifaceted nature of AOSD, carefully designed clinical studies aimed to associate distinct clinical phenotypes with specific pathogenetic pathways would allow the designation of tailored therapies for distinct disease aspects. 

## Figures and Tables

**Table 1:** Cytokines involved in the pathogenesis of AOSD and their potential associations with clinical/serological manifestations and disease activity. MAS: Macrophage activation syndrome, CRP: C-Reactive Protein, AOSD: Adult Onset Still's Disease.

Cytokine	Clinical-serological associations
Tumor necrosis factor-*α* (TNF-*α*)	TNF-*α* serum concentration is not associated with disease activity [8, 26]. Levels of TNF receptor (TNF-R) correlate with serum levels of CRP
Interleukin-1*β* (IL-1*β*)	Potential marker for disease activity and useful for monitoring the efficacy of treatment [27, 28]
Soluble interleukin-2 receptor (sIL-2R)	Marker of disease activity [8, 29]
Interleukin-6 (IL-6)	Elevation of CRP, Hyperferritinaemia, Salmon-colored rash
	Hepatosplenomegaly, marker of disease activity [29–31]
Interleukin-8 (IL-8)	Prediction of persistent arthritis but not of disease activity [30]
Interleukin-17 (IL-17)	Elevated in active disease and normalization with the appropriate therapy [20]
Interleukin-18 (IL-18)	Elevation of CRP, hyperferritinaemia, neutrophilia, AOSD-related hepatitis
	Marker of disease severity and response to corticosteroids, MAS [30, 32–36]
Interferon-*γ* (IFN-*γ*)	Elevated levels in AOSD patients but no correlation with disease activity [26, 29]

**Table 2:** Safety and efficacy of biological agents used in the treatment of AOSD/SJIA in several studies. No: number, Pts: patients, DMARDs: Disease modifying antirheumatic drugs, CSs: corticosteroids, CRP: C-reactive protein, ESR: erythrocyte sedimentation rate, SJIA: systemic juvenile idiopathic arthritis, CR: complete response, PR: partial response, NR: Nonresponders.

Author	No. of pts	Therapeutic regimen	Duration of treatment (months)	Clinical/serological effects	Adverse events
Kraetsch et al. (2001) [52]	6 AOSD pts	Infliximab + DMARDs + CSs	5–28	Resolution of systemic features/Normalization of inflammatory markers	Infusion reactions
Husni et al. (2002) [53]	12 AOSD pts	Etanercept + MTX + CSs ± NSAIDs	6	Two pts with systemic features withdrew (flare). Approximately two thirds improvement of arthritic complaints	Injection-site reactions, upper respiratory tract illness, rash, diarrhea, sinusitis
Dechant et al. (2004) [54]	8 AOSD pts	Infliximab + DMARDs + CSs	1–5	Improvement of systemic features and serological markers	Infusion reactions
Kokkinos et al. (2004) [55]	4 AOSD pts	Infliximab + MTX + CSs	3.5–18	Remission of systemic features Normalization of inflammatory markers and liver function tests	None reported
Fautrel et al. (2005) [56]	20 AOSD pts	Infliximab and/or etanercept + MTX + CSs	11 for etanercept/9 for infliximab	Remission: 5 pts/Failure: 4 pts/the rest: partial response	Recurrent bronchitis, lupus rash, optic neuritis, cardiac failure, thigh abscess, rash
Fitzgerald et al. (2005) [57]	4 AOSD pts	Anakinra + MTX + CSs	6–19	Rapid resolution of clinical and inflammatory markers in all pts	Viral pneumonia, idiopathic pulmonary hypertension, shingles, flu-like syndrome
Woo et al. (2005) [58]	18 SJIA pts	Tocilizumab + CSs ± MTX	1-2	Eleven patients achieved ACR 30 responses, eight achieved ≥50% ACR responses	Oral herpes simple, low lymphocytic levels, and transient increases in ALT
Kötter et al. (2007) [28]	4 AOSD pts	Anakinra + CSs + DMARDs	12–44	Rapid resolution of clinical and inflammatory markers in all pts within days. Tapering of CS therapy could not be achieved.	Injection-site erythema that improved within 6 weeks of therapy
Kalliolias et al. (2007) [59]	4 AOSD pts	Anakinra ± CSs	5–17	Normalization of clinical (within hours) and inflammatory markers (within 2–4 weeks) along with liver enzymes (within 3 weeks) in all pts. Rapid tapering of CS therapy.	Self-limited injection-site erythema
Lequerré et al. (2008) [60]	15 AOSD and 20 SJIA pts	Anakinra + CSs ± DMARDs	11–27	AOSD → CR:9 pts, PR:2 pts, NR:2 pts, Intolerance: 2 pts. Anakinra was stopped in 2 pts due to adverse skin reactions SJIA → CR: 6 pts, PR: 4 pts NR: 10 pts (2 at 2 months). 1 patient who initially achieved a CR developed visceral leishmaniasis and anakinra was stopped.	AOSD: bronchitis, uncomplicated hepatitis A, varicella, cutaneous infections, osteonecrosis of the femoral hip (attributed to CS treatment), local pain and injection-site reactions. SJIA: rhinopharyngitis nonextensive labial herpes and visceral leishmaniasis
Laskari et al. (2011) [61]	25 AOSD pts	Anakinra + DMARDs	1.5–71	Complete clinical response in 84% of pts, partial in 12%/Complete laboratory response in 80% of pts	Severe urticarial reaction, various infections, local injection reaction
Nigrovic et al. (2011) [62]	46 SJIA pts	Anakinra + DMARDs + CSs	14.5	Systemic features resolved within 1 month in >95% of pts/persistence of active arthritis in 11%. CRP and ferritin normalized within 1 month ( >80% of pts)	Injection site reactions, eosinophilic hepatitis, mild asymptomatic neutropenia, and elevation of liver enzymes
Puéchal et al. (2011) [63]	14 AOSD pts	Tocilizumab + DMARDs + CSs	6	Good EULAR response in 64% of pts at 3 months/EULAR remission in 57% at 6 months/Inflammatory markers improved	Necrotizing angiodermatitis, chest pain, mild hyperlipidemia, elevation of liver enzymes
Ruperto et al. (2012) [64]	23 SJIA pts	Canakinumab + CSs	24	60% responders according to the adapted ACR Pediatric 50 criteria and 4 patients inactive by day 15	Two severe possibly related to the study drug: Epstein-Barr virus infection and hematoma, prolonged activated partial thromboplastin time, gastroenteritis, and syncope
Kontzias and Efthimiou (2012) [65]	2 AOSD pts	Canakinumab + CSs ± MTX	6–12	Normalization of inflammatory markers and remission of both systemic and arthritic manifestations	None reported
